# A Cell-Based ELISA for High-Throughput Screening of Inhibitors Blocking Influenza Virus Attachment to the Subtype-Independent α2,6-Sialic Acid Receptor

**DOI:** 10.3390/ijms27209205

**Published:** 2026-10-16

**Authors:** Keyu Guo, Jingshuai Wu, Yaru Liu, Jiahui Luo, Weilian Jiang, Chenyin Wang, Ren Sheng, Shunwang Li, Xiuyong Fan, Yexiang Wu, Yanni Xu, Shuyi Si, Yongxin Zhang, Jing Zhang

**Affiliations:** Beijing Key Laboratory of Technology and Application for Anti-Infective New Drugs Research and Development, Institute of Medicinal Biotechnology, Chinese Academy of Medical Sciences & Peking Union Medical College, Beijing 100050, China

**Keywords:** influenza viruses, high-throughput screening model, entry inhibitors, hemagglutinin, Sambucus nigra agglutinin (SNA), α-2,6-sialic acid, subtype-independent receptor recognition

## Abstract

Rising drug resistance underscores the urgent need for novel anti-influenza strategies. Targeting the hemagglutinin (HA)–sialic acid receptor interaction offers a promising broad-spectrum approach, yet progress is impeded by the lack of screening models that report the receptor-recognition step in a subtype-independent manner. Herein, we established a robust cell-based ELISA platform for high-throughput screening (HTS) that operates on the subtype-independent receptor-recognition step. The model utilizes ST6GAL1-overexpressing MDCK cells to enrich human-type α2,6-sialic acid receptors and employs horseradish peroxidase (HRP)-conjugated Sambucus nigra lectin (SNA) as a functional, though not structural, surrogate for the receptor-recognition step, yielding a high Z’ factor of 0.78. Given that SNA is a lectin rather than HA, the assay cannot by itself distinguish HA-specific blockade from membrane-mediated or virucidal effects. Orthogonal validation is therefore required for every hit. Screening a library of 2600 compounds identified two hits, Hypericin and protoporphyrin IX (PPIX), which effectively blocked SNA–receptor recognition with IC_50_ values of 18.39 ± 6.23 μM and 9.77 ± 3.05 μM, respectively. An orthogonal flow cytometry assay with FITC-conjugated SNA reproduced this inhibition on intact cells in a dose-dependent manner (IC_50_ = 17.68 ± 9.54 μM for Hypericin and 25.31 ± 19.47 μM for PPIX), confirming that the inhibition is not an artifact of the colorimetric readout. Hemagglutination inhibition (HA) assays with distinct pre-incubation strategies indicated that both compounds act on the viral particle rather than on the host receptor. Surface plasmon resonance (SPR) and molecular docking studies further indicated direct, dose-dependent but low-affinity binding to HA proteins. Notably, cytopathic effect (CPE) assays demonstrated their potent antiviral efficacy across H1N1 and H3N2 subtypes, with PPIX exhibiting exceptionally potent activity against H3N2 (EC_50_ = 0.50 ± 0.17 μM). Taken together, these data identify Hypericin and PPIX as viral-side attachment inhibitors with weak HA binding and multimodal antiviral activity. Because neither a virucidal nor a fusion-blocking control was included, the relative contribution of HA engagement to their overall antiviral effect remains to be established. In conclusion, this BSL-1-compatible ELISA platform, which probes the subtype-independent receptor-recognition step, provides an efficient tool for discovering broad-spectrum influenza entry inhibitors, and the identified hits offer novel pharmacological scaffolds for further structural optimization.

## 1. Introduction

Seasonal influenza epidemics impose a substantial economic encumbrance on global healthcare systems and society, transcending direct medical costs to include significant indirect losses from workplace absenteeism and reduced productivity [1]. Moreover, the seasonal nature of influenza outbreaks also creates a recurrent challenge for healthcare systems and places additional strain on healthcare resources and personnel [2]. Despite the widespread availability of vaccines, the efficacy of immunization is frequently compromised by antigenic drift and shift, requiring annual reformulations that do not always perfectly match circulating strains, thus limiting their protective impact across diverse demographics [3]. Concurrently, the therapeutic landscape is increasingly precarious. First-line antivirals, such as M2 ion channel blockers and neuraminidase inhibitors, face diminishing utility due to the rapid emergence of resistant viral strains [4]. This escalating resistance highlights an urgent need for novel antiviral agents that target conserved viral components with a higher genetic barrier to resistance, thereby securing a more sustainable long-term defense strategy.

The initial interaction between the viral hemagglutinin (HA) and host sialic acid receptors represents a critical bottleneck in the viral life cycle, serving as the gateway for viral entry and subsequent dissemination [5]. Pharmacologically disrupting HA–receptor engagement offers a dual benefit. Small-molecule inhibitors that bind the HA receptor-binding site can block HA–receptor interaction and thereby prevent viral attachment to new host cells [6]. In addition, HA-interacting antibodies disrupt HA trimers, limiting cell-to-cell spread of progeny virions in cultures [7]. However, developing broad-spectrum inhibitors targeting this step has been historically challenging due to the hypervariability of the HA globular head domain across different subtypes (e.g., H1, H3, H5) [8]. Traditional screening methodologies that rely on specific HA subtypes often yield narrow-spectrum inhibitors, failing to address the threat of antigenic variation. Consequently, there is a pressing demand for a screening paradigm that bypasses viral variability by focusing on the conserved host receptor machinery.

Current methodologies for identifying entry inhibitors suffer from distinct operational and safety drawbacks. Live virus-based assays, while physiologically relevant, require high-containment biosafety level 2 or 3 (BSL-2/3) facilities, which are resource-intensive and unsuitable for high-throughput screening (HTS) [9]. Although pseudovirus systems mitigate biosafety risks, they remain complex and often fail to isolate the receptor-binding event from downstream fusion processes. Furthermore, conventional hemagglutination inhibition (HAI) assays are semi-quantitative, prone to subjective interpretation, and lack the scalability required for screening large compound libraries [10,11]. There is, therefore, a critical unmet need for a robust, BSL-1-compatible platform that reports the subtype-independent receptor-recognition step and can quantitatively assess the inhibition of HA–receptor recognition in a high-throughput format.

To circumvent the barrier of viral variability, we hypothesized that targeting the conserved host receptor, α-2,6-linked sialic acid, the primary binding site for human-adapted influenza viruses, could enable the discovery of influenza entry inhibitors through a subtype-independent readout of the receptor-recognition step. Herein, we report the development of a novel cell-based enzyme-linked immunosorbent assay (ELISA) HTS platform. This model utilizes horseradish peroxidase–conjugated Sambucus nigra lectin (HRP-SNA), a stable and universal probe that specifically recognizes α-2,6-linked sialic acids, as a functional surrogate for the diverse and variable HA subtypes [12,13]. By integrating this probe with MDCK cells engineered to overexpress ST6GAL1 (α-2,6-sialyltransferase) to enrich surface receptors, we established a standardized, colorimetric assay that operates under BSL-1 conditions. This platform allows for the direct, quantitative measurement of compound interference at the virus–host interface via a simple optical density (OD_450_) readout, providing an efficient tool for accelerating the discovery of broad-spectrum influenza entry inhibitors.

## 2. Results

### 2.1. Establishment and Principle of the Cell-Based ELISA Screening Model

**Figure 1:**
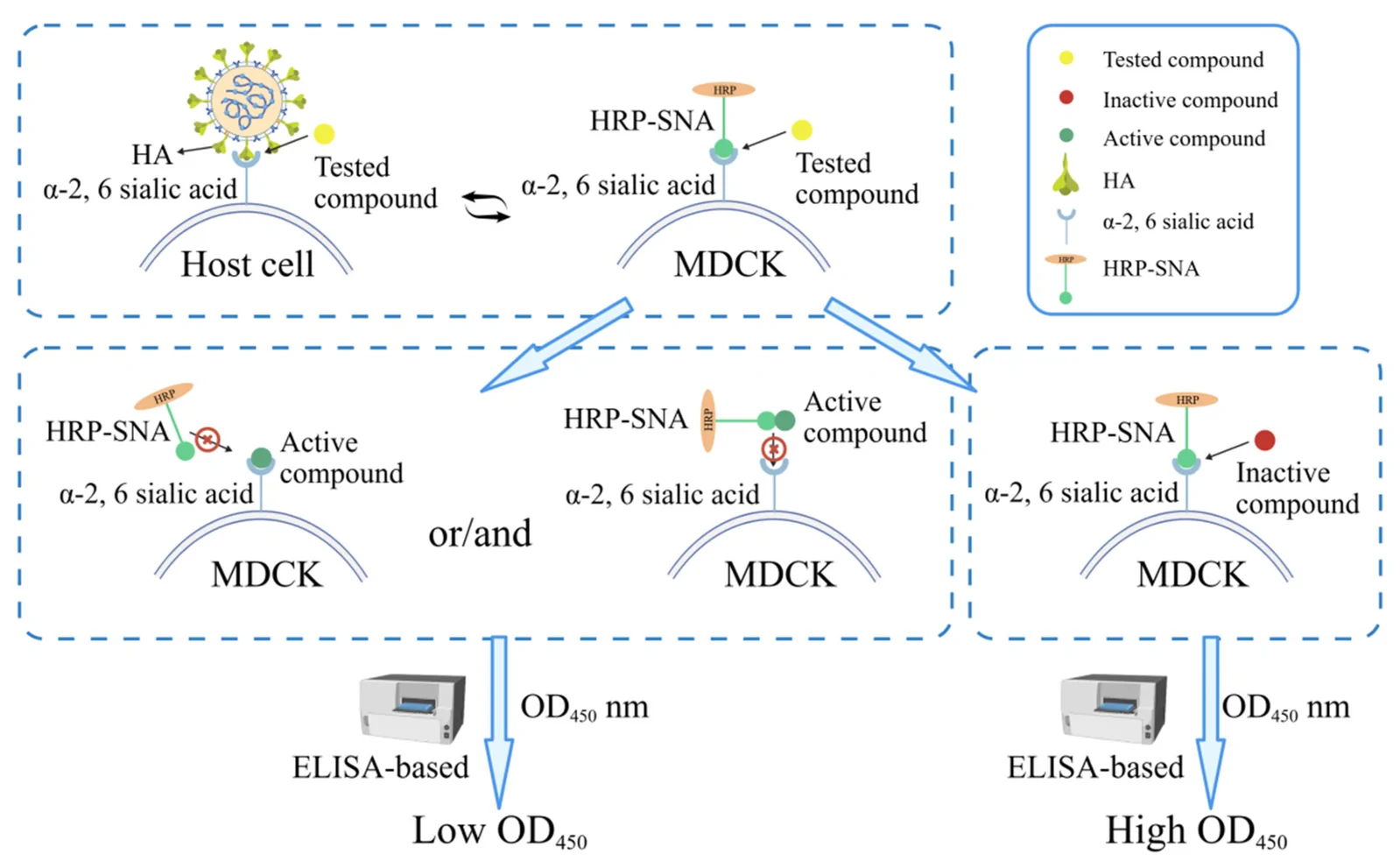
Created with BioGDP.com [16]. Schematic representation of the cell-based ELISA screening model, which targets the subtype-independent receptor-recognition step. The model utilizes MDCK cells overexpressing ST6GAL1 to enrich surface α2,6-sialic acid receptors. HRP-conjugated Sambucus nigra lectin (HRP-SNA) serves as a stable surrogate for the viral hemagglutinin (HA) protein. In the absence of an inhibitor, HRP-SNA binds to the receptors, and subsequent TMB substrate reaction yields a high OD_450_ signal. An effective entry inhibitor blocks the SNA–receptor interaction, leading to a decrease in bound HRP and a reduced OD_450_ readout.

To address the challenge of screening for broad-spectrum influenza inhibitors targeting the highly variable HA protein, we developed a cell-based ELISA model that targets the subtype-independent receptor-recognition step. The construction of this model is based on the principle that SNA specifically binds to α-2,6-linked sialic acid [12,13], the primary host receptor for human-adapted influenza viruses [14]. Consequently, HRP-SNA was utilized as a stable surrogate for the viral HA protein, reporting the receptor-recognition step rather than HA binding per se. In this system, MDCK cells stably overexpressing human ST6GAL1 were seeded in 96-well plates to enrich surface α-2,6-sialic acid receptors. After incubation with HRP-SNA, the unbound probe was washed away, and the bound HRP was quantified using TMB substrate by measuring absorbance at OD_450_ [15]. The presence of an inhibitor blocking the interaction between SNA and the receptor results in a decrease in bound HRP, thereby reducing the OD_450_ signal (Figure 1).

### 2.2. Comparative Binding of Influenza HA and SNA to the Same Receptor

**Figure 2:**
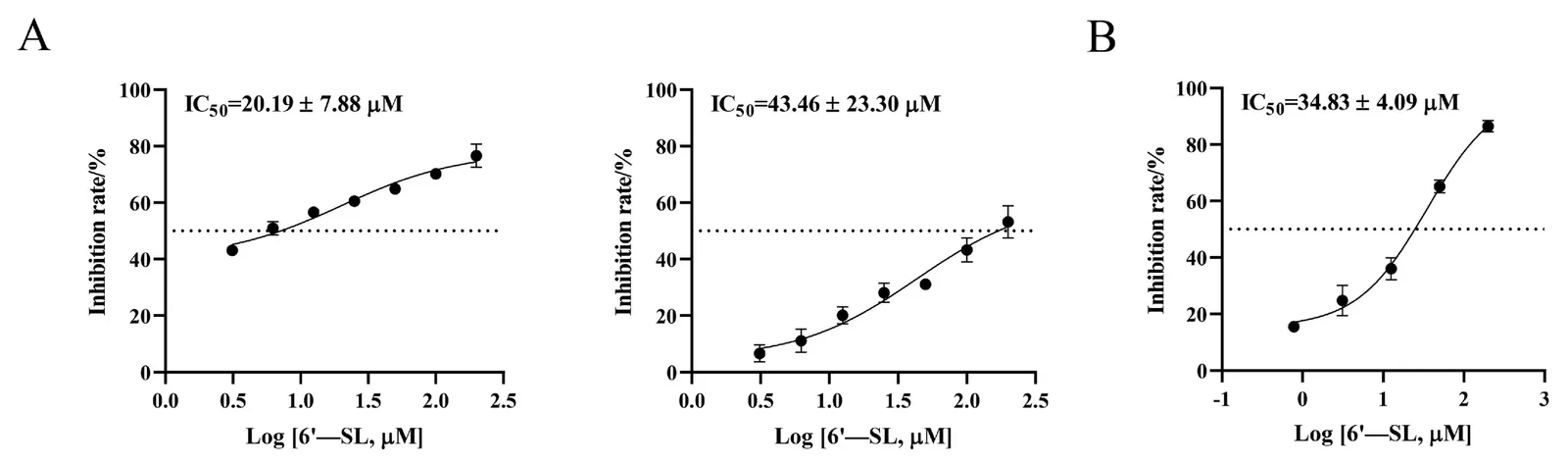
Binding of HA and SNA to a shared receptor. (**A**) Recombinant H1N1 (A/Puerto Rico/8/1934), H3N2 (A/Brisbane/10/2007) HA and HRP-SNA were incubated in parallel with fixed MDCK-ST6GAL1 monolayers. Bound HA was detected with subtype-matched HRP-conjugated anti-HA antibodies, and bound SNA through its conjugated HRP. (**B**) In a parallel competition arm, 6’-SL (0.78–200 μM) dose-dependently inhibited the binding of all three probes. Data are mean ± SD, *n* = 3 triplicate wells.

To verify that the SNA-based readout reports the same receptor pool that influenza HA uses, recombinant H1N1 HA (A/Puerto Rico/8/1934), H3N2 HA (A/Brisbane/10/2007) and HRP-SNA were applied in parallel to replicate monolayers of MDCK-ST6GAL1 cells as described in Section 4.2. All three probes bound the fixed monolayers in a concentration-dependent manner, whereas probe-free control wells gave no detectable signal. Because the probes differ in working concentration and detection chemistry, their absolute OD_450_ values were not compared directly. Binding was instead compared through a common competitor: 6′-SL, the trisaccharide representing the α-2,6-linked sialic acid receptor motif recognized by the virus, which also served as the positive control inhibitor of the screen [17]. 6′-SL dose-dependently inhibited the binding of all three probes, with IC_50_ values of 20.19 ± 7.88 μM for H1N1 HA, 43.46 ± 23.30 μM for H3N2 HA and 34.80 ± 4.09 μM for HRP-SNA (Figure 2). These results indicate that both HA subtypes and SNA engage an overlapping α2,6-sialylated receptor population on the same cell surface, supporting the use of HRP-SNA as a functional surrogate for HA in this screening model. This value is in line with the low intrinsic affinity of monovalent sialosides, which is well documented for unconjugated sialic-acid derivatives, further confirming the reliability of our screening model [18].

### 2.3. Optimization of the Cell-Based ELISA Screening Model

**Figure 3:**
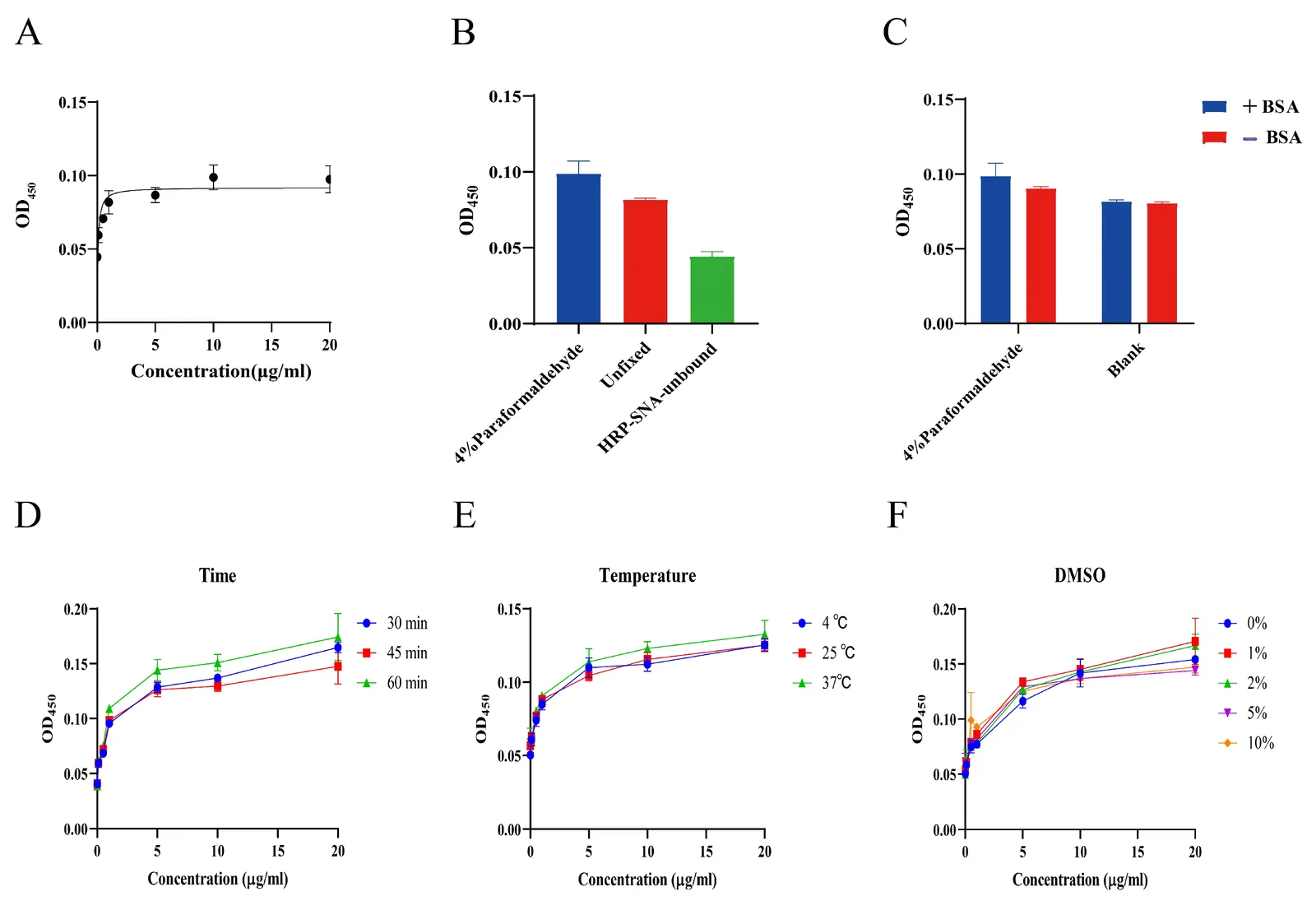
Optimization of the cell-based ELISA screening model. Key assay parameters were systematically optimized to ensure sensitivity and stability. (**A**) Titration of HRP-SNA probe concentration (0.1 to 20 μg/mL), with 1 μg/mL selected as optimal. (**B**) Effect of cell fixation with 4% PFA versus unfixed controls. (**C**) Effect of blocking with 5% BSA versus unblocked conditions. (**D**) Optimization of HRP-SNA incubation time (30 min, 45 min, 60 min). (**E**) Optimization of incubation temperature (4 °C, 25 °C, 37 °C). (**F**) DMSO tolerance assessment (0% to 10%). Data are presented as mean ± SD of *n* = 3 independent biological replicates.

To establish a robust high-throughput screening (HTS) model for influenza virus entry inhibitors, we systematically optimized the key experimental parameters. The feasibility of this approach was first validated by titrating the HRP-SNA probe. The results demonstrated that OD_450_ values increased significantly in a dose-dependent manner with increasing concentrations of HRP-SNA, reaching a plateau between 1 and 5 μg/mL. To ensure a strong signal while minimizing reagent consumption, 1 μg/mL was selected as the optimal screening concentration (Figure 3A). Following this, other critical parameters were systematically optimized. Comparison of fixed (4% PFA) and unfixed cells revealed no significant difference in HRP-SNA binding capacity. However, 4% PFA fixation was adopted to ensure biosafety and operational stability (Figure 3B). Blocking with 5% BSA produced OD_450_ values comparable to those of unblocked wells (Figure 3C), so this step was therefore omitted to streamline the screening procedure. Furthermore, optimal binding kinetics were achieved with an incubation time of 60 min (which provided a significantly stronger signal compared to 30 min), and incubation at 37 °C yielded the highest OD_450_ values compared to 25 °C or 4 °C (Figure 3D,E). Finally, we assessed the solvent tolerance of the system, confirming that the assay signal remained stable at DMSO concentrations of 2% or below, indicating compatibility with standard small-molecule libraries (Figure 3F).

In summary, the final optimized conditions for the HTS platform were established as follows: MDCK cells seeded at 1 × 10^5^ cells/well, fixed with 4% PFA without blocking, incubation with 1 μg/mL HRP-SNA at 37 °C for 60 min, and a maximum DMSO concentration of 2%.

### 2.4. Model Robustness Assessment

**Figure 4:**
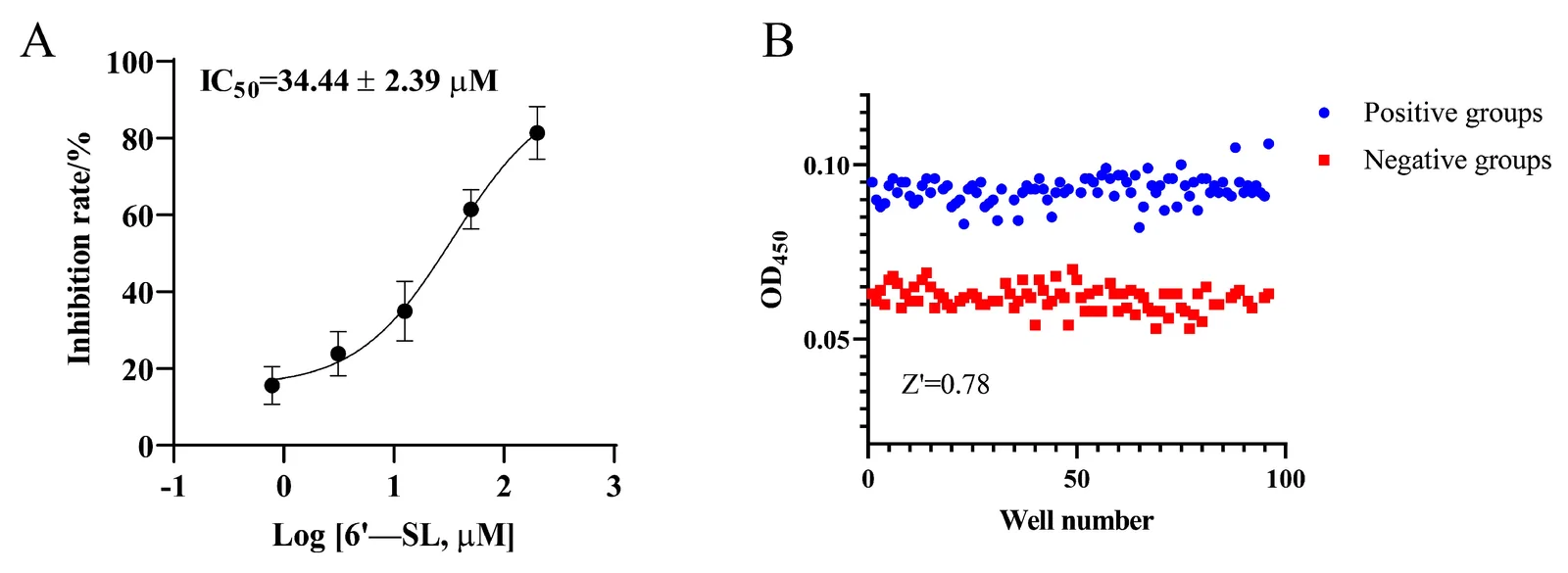
Model robustness assessment. (**A**) Inhibitory dose–response curves for the positive control 6′-SL against HRP-SNA binding to fixed MDCK-ST6GAL1 monolayers, determined in three independent concentration series (0.78–200 μM). (**B**) Scatter plot illustrating the Z’ factor analysis.

As the assay is intended for routine screening, the reproducibility of its potency readout across independent runs was assessed first. The IC_50_ of the positive control 6′-SL was determined in three independent concentration series, each performed on a different day with independently prepared reagents, and the three series gave a combined IC_50_ of 34.44 ± 2.39 μM, corresponding to an inter-assay CV of 6.95% (Figure 4A). The reliability of the optimized assay for large-scale screening was confirmed by calculating the Z’ factor. Based on the optimized parameters, the Z’ factor was determined to be 0.78 (Figure 4B). This high Z’ value exceeds the standard threshold of 0.5 for HTS assays [19], demonstrating that the model possesses excellent robustness, low variability, and high reproducibility.

### 2.5. Primary Screening and Identification of Active Inhibitors

**Figure 5:**
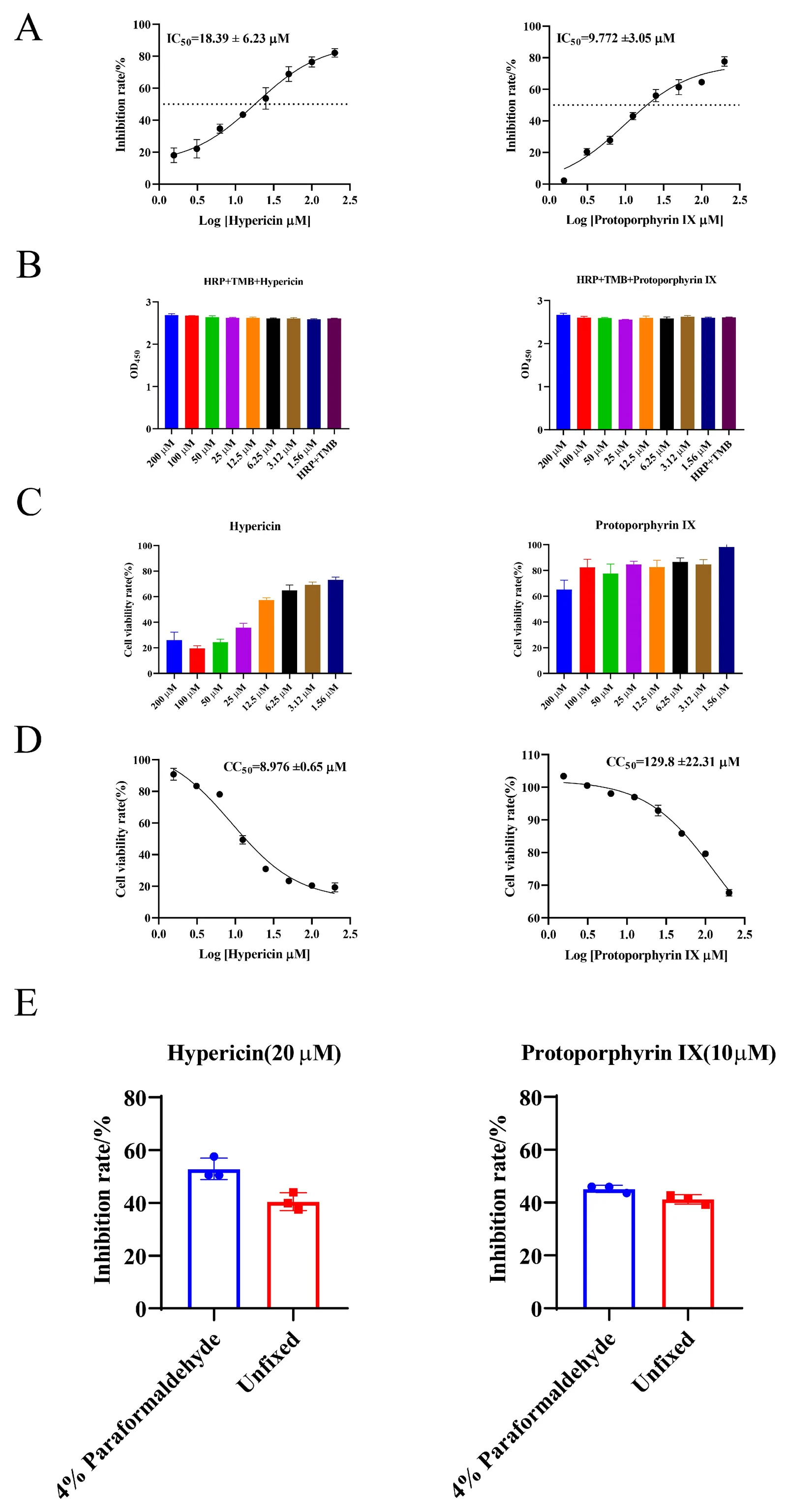
Primary screening and validation of active inhibitors. (**A**) Dose–response curves of the hit compounds Hypericin and Protoporphyrin IX (PPIX) in the cell-based ELISA, inhibiting SNA–receptor interaction with IC_50_ values of 18.39 ± 6.23 μM and 9.77 ± 3.05 μM, respectively. (**B**) Assessment of potential compound interference with the detection system. OD_450_ readings of reactions containing compounds were comparable to the HRP-SNA control. (**C**) Short-term (2 h) cytotoxicity evaluation of Hypericin and PPIX, ruling out false positives due to cell detachment. (**D**) Long-term (48 h) cytotoxicity evaluation using the CCK-8 assay, revealing distinct safety profiles for the two compounds. Data are presented as mean ± SD of *n* = 3 independent biological replicates. (**E**) Inhibition of HRP-SNA binding by Hypericin (20 μM) and PPIX (10 μM) in fixed versus unfixed MDCK-ST6GAL1 monolayers.

Using the validated cell-based ELISA model, we performed a primary high-throughput screen of a library containing 2600 compounds at a single concentration of 25 μM. This initial screen identified four compounds that exhibited an inhibition rate greater than 50% against HRP-SNA binding. To eliminate false positives and confirm their inhibitory activity, these four hits were subjected to secondary screening involving dose–response curve analysis. Among the candidates, only two compounds, Hypericin and PPIX, displayed a clear dose-dependent inhibition of the SNA–receptor interaction. The calculated IC_50_ values were 18.39 ± 6.23 μM for Hypericin and 9.77 ± 3.05 μM for PPIX, respectively (Figure 5A).

Although the cell-based ELISA model is highly specific for receptor recognition, there is a potential risk of identifying false-positive compounds that inhibit the HRP enzyme directly or otherwise interfere with the detection system. Therefore, we performed validation experiments to exclude these interferences for the identified hits. We incubated different concentrations of the compounds with the HRP-SNA probe and TMB substrate. The results showed that the OD_450_ readings of the reaction system containing the compounds were comparable to those of the control group (HRP-SNA and TMB without compounds) (Figure 5B). This indicates that neither Hypericin nor PPIX inhibits the enzymatic activity of HRP. Collectively, these results confirm that the inhibitory effects observed in the screening assay are attributable to the specific blockade of the SNA–receptor interaction, rather than artifacts of the detection system.

Considering that the compounds are incubated with cells for approximately 1 h, and then with HRP-SNA for an additional 1 h during the ELISA assay, we first evaluated their cytotoxicity within this specific detection window (Figure 5C). The results demonstrated that neither Hypericin nor PPIX exhibited any observable cytotoxicity to the cells during this 2 h incubation period. This short-term assessment ruled out the possibility that the compounds reduced the OD_450_ readings by inducing cellular toxicity and subsequent cell detachment, rather than genuinely blocking the SNA–receptor interaction. To comprehensively evaluate their safety profiles, subsequent cytotoxicity evaluation using the CCK-8 assay was conducted over a 48 h exposure period, which revealed distinct safety profiles for the two hits. Hypericin demonstrated certain cytotoxicity, with cell viability dropping below 50% at the screening concentration of 25 μM (Figure 5D). In contrast, PPIX showed a favorable safety profile, with cell viability remaining above 50% even at a high concentration of 200 μM (Figure 5D). We next asked whether the PFA fixation step required by the optimized protocol could itself bias the apparent potency of the hits. At concentrations near their respective IC_50_ values (Hypericin, 20 μM; PPIX, 10 μM), inhibition of SNA binding was comparable in fixed and unfixed MDCK-ST6GAL1 monolayers (Figure 5E), indicating that fixation did not affect the assay outcome.

### 2.6. Fixation-Independence and Orthogonal Validation of the Screening Hits by Flow Cytometry

**Figure 6:**
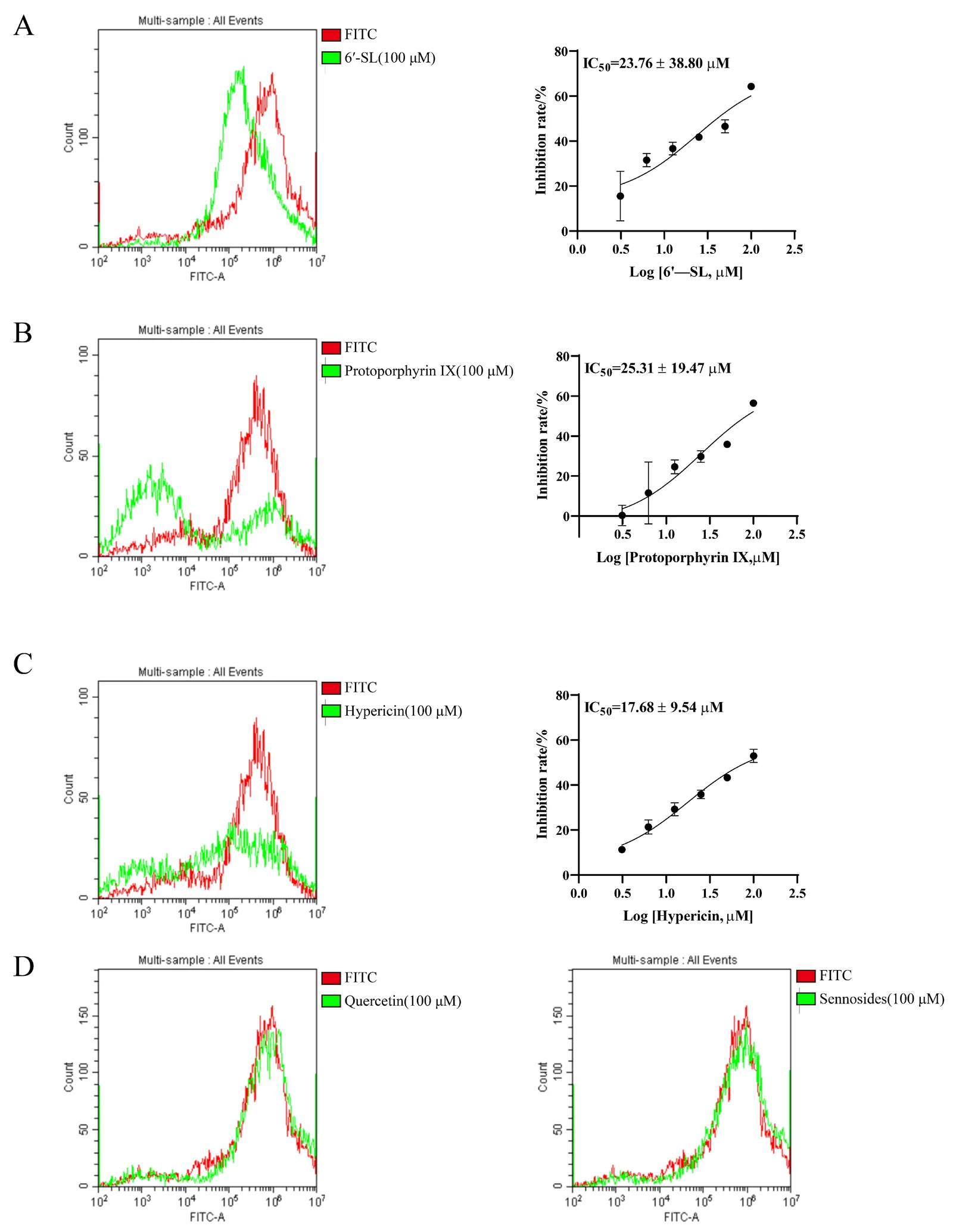
Orthogonal validation of the screening hits by flow cytometry. (**A**–**C**) Representative flow cytometry histograms of FITC-SNA binding to MDCK-ST6GAL1 cells (left) and the corresponding dose–response curves fitted to the integrated mean fluorescence intensity (iMFI) (right) for the positive control 6′—SL (**A**), PPIX (**B**) and Hypericin (**C**). (**D**) Representative histograms of cells treated with two compounds that scored negative in the primary screen. Data are presented as mean ± SD of *n* = 3 independent biological replicates.

Having confirmed that PFA fixation does not alter the inhibition measured for the hits, we went on to ask whether the two ELISA-derived hits represent genuine inhibition of receptor–ligand binding at the cellular level, and performed an orthogonal flow cytometry assay using FITC-conjugated SNA. Both Hypericin and PPIX inhibited FITC-SNA binding to MDCK-ST6GAL1 cells in a dose-dependent manner, with IC_50_ values of 17.68 ± 9.54 μM and 25.31 ± 19.47 μM, respectively (Figure 6B,C), in agreement with the ELISA-derived values (18.39 ± 6.23 μM and 9.77 ± 3.05 μM). The discrepancy for PPIX fell within its comparatively wide standard deviation. By contrast, two compounds (Quercetin and Sennosides) that exhibited less than 20% inhibition in the primary screen produced almost no inhibition in the flow cytometry assay (<10% at 100 μM; Figure 6D), whereas the positive control 6′-SL reproduced its dose-dependent inhibition in the flow format (IC_50_: 23.76 ± 38.80 μM) (Figure 6A), consistent with its ELISA-derived IC_50_ (34.44 ± 2.39 μM). Control samples acquired in the absence of probe or in the absence of compound excluded any contribution of the intrinsic fluorescence of the two macrocyclic compounds to the measured signal. Collectively, this cell population-based analysis shows that the hits identified by the ELISA correspond to authentic inhibition of the receptor-binding event at the cellular level.

### 2.7. Hemagglutination Inhibition (HAI) Assays

To investigate whether the identified compounds could inhibit the biological interaction between influenza virus HA and host receptors, we performed HAI assays using Influenza A H1N1 (A/Puerto Rico/8/1934) and Influenza A H3N2 (A/Aichi/2/1968) subtypes. To elucidate their mechanism of action, we employed two distinct pre-incubation strategies: pre-incubating the compounds with the influenza virus (HA) prior to the addition of GPRBCs, or pre-incubating the compounds with GPRBCs prior to the addition of the influenza virus (HA).

**Figure 7:**
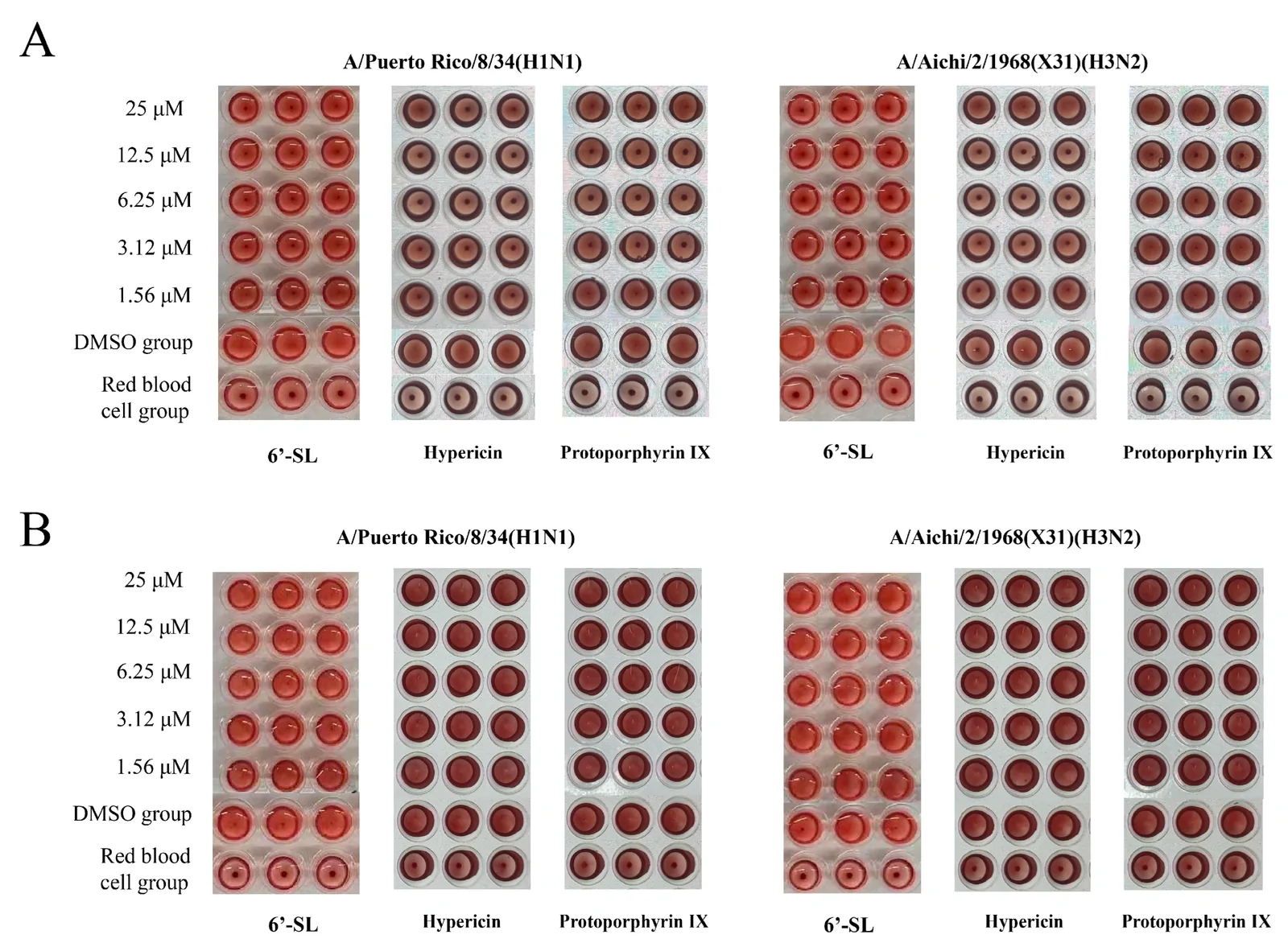
Hemagglutination inhibition (HAI) assays. (**A**) Inhibition of viral HA-mediated agglutination when compounds were pre-incubated with the influenza virus (HA) prior to the addition of guinea pig red blood cells (GPRBCs). 6’-SL, Hypericin and PPIX effectively inhibited agglutination against H1N1 and H3N2 subtypes in a dose-dependent manner. (**B**) Hemagglutination results when compounds were pre-incubated with GPRBCs before the addition of the influenza virus (HA). No inhibition was observed for either compound, indicating they do not bind to host sialic acid receptors and specifically target the viral HA protein.

When the compounds were pre-incubated with the influenza virus (HA), both Hypericin and PPIX effectively inhibited viral HA-mediated agglutination. Notably, both compounds exhibited more potent inhibitory activity against the H1N1 subtype than H3N2. For H1N1, Hypericin inhibited hemagglutination at concentrations ranging from 1.56 to 25 μM, while PPIX was active from 3.12 to 25 μM (Figure 7A). For H3N2, Hypericin displayed inhibitory activity between 6.25 and 25 μM, whereas PPIX inhibited agglutination between 6.25 and 12.5 μM (Figure 7A). The soluble receptor analogue 6’-SL, which likewise binds the viral HA rather than host receptors (Figure 7A,B), inhibited agglutination of both subtypes in a dose-dependent manner (Figure 7A).

Conversely, when the compounds were pre-incubated with GPRBCs before the addition of the influenza virus (HA), no inhibition of hemagglutination was observed for either compound (Figure 7B). The same result was obtained for 6’-SL. This indicates that the compounds did not interfere with the binding between influenza HA and the GPRBCs, thereby arguing against the possibility that the compounds exert their inhibitory effects by binding to the host sialic acid receptors. Collectively, these results indicate that the compounds act on the viral side of the interaction rather than on the host receptor. HAI reports only the loss of virion bridging to erythrocytes and cannot by itself distinguish blockade of the HA receptor-binding site from virion damage or from interference with a later step such as fusion. The observation is therefore interpreted here as a viral-side attachment effect, one that requires the orthogonal confirmation provided by flow cytometry, SPR and CPE assays.

### 2.8. Binding Affinity Analysis via SPR

**Figure 8:**
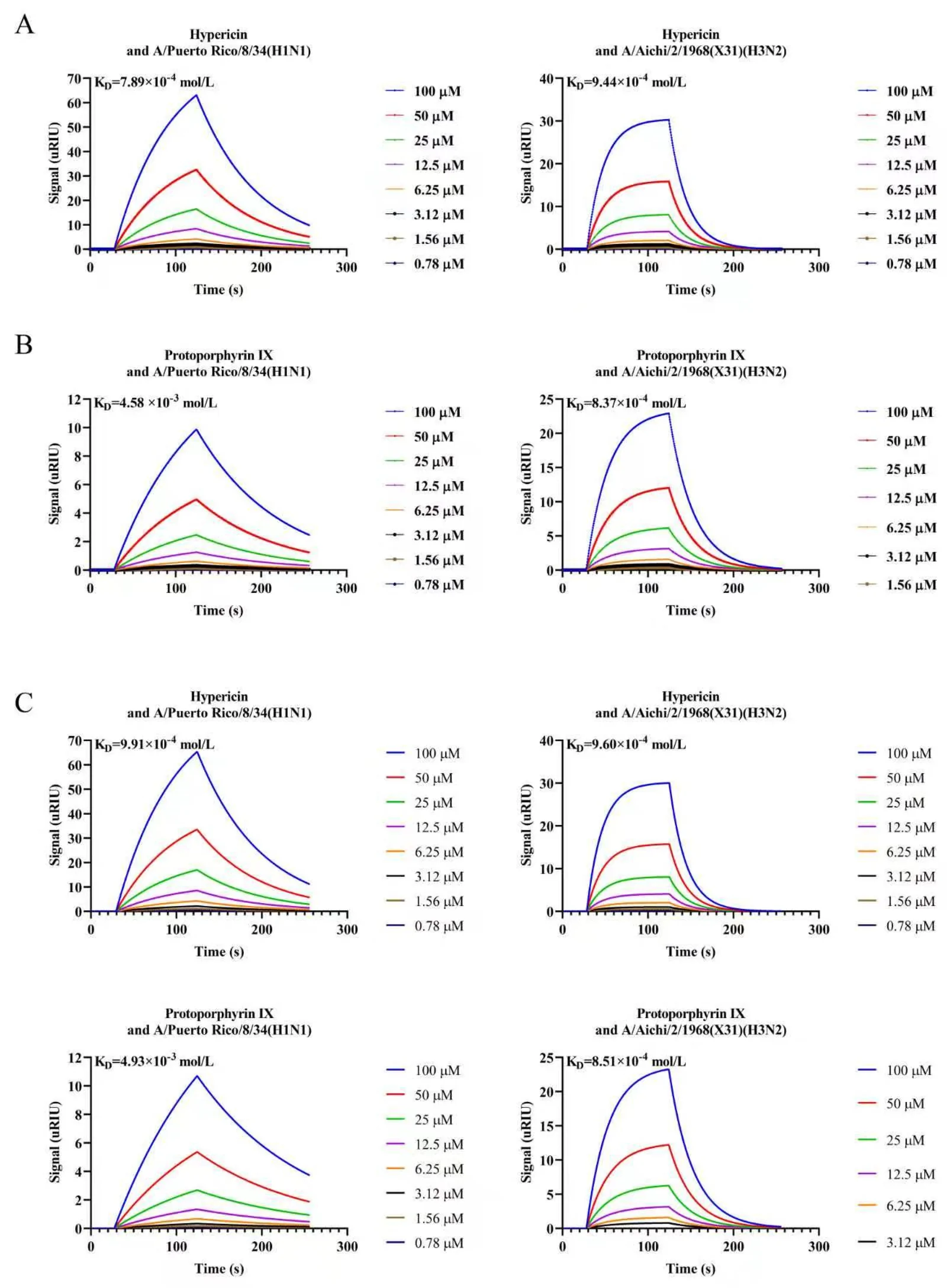
Binding affinity analysis via Surface Plasmon Resonance (SPR). Recombinant HA proteins from H1N1 and H3N2 subtypes were immobilized on a sensor chip, and different concentrations of compounds were flowed over the surface. (**A**) SPR sensorgrams of Hypericin binding to H1N1 and H3N2 HA proteins (K*_D_* = 7.89 × 10^−4^ mol/L and 9.44 × 10^−4^ mol/L, respectively). (**B**) SPR sensorgrams of PPIX binding to H1N1 and H3N2 HA proteins (K*_D_* = 4.58 × 10^−3^ mol/L and 8.37 × 10^−4^ mol/L, respectively). (**C**) SPR reference-channel control. Equal amounts of BSA were immobilized on a parallel CM5 channel, and the identical concentration series of Hypericin and PPIX were injected over the BSA surface.

To further validate the direct interaction between the hit compounds and the viral target, SPR analysis was conducted. Recombinant HA proteins from Influenza A H1N1 (A/Puerto Rico/8/1934) and Influenza A H3N2 (A/Aichi/2/1968) subtypes were immobilized on the sensor chip, and different concentrations of Hypericin and PPIX were flowed over the surface. The SPR analysis confirmed that both compounds bind directly to the HA proteins of both H1N1 and H3N2 subtypes in a dose-dependent manner. The K*_D_* for Hypericin were determined to be 7.89 × 10^−4^ mol/L for H1N1 and 9.44 × 10^−4^ mol/L for H3N2 (Figure 8A), while PPIX exhibited K*_D_* values of 4.58 × 10^−3^ mol/L for H1N1 and 8.37 × 10^−4^ mol/L for H3N2 (Figure 8B). Neither compound showed appreciable binding to the equal-amount BSA reference channel (Figure 8C), and after subtracting this non-specific signal, the K*_D_* values were fitted from the specific HA-binding curves, confirming that the interaction is HA-specific rather than a surface artifact. This direct binding evidence corroborates the results from the HAI assays and the cell-based ELISA, supporting the classification of Hypericin and PPIX as viral-side attachment inhibitors with weak HA binding and multimodal antiviral activity.

### 2.9. Molecular Docking Predicts Binding to Conserved HA Sites

**Figure 9:**
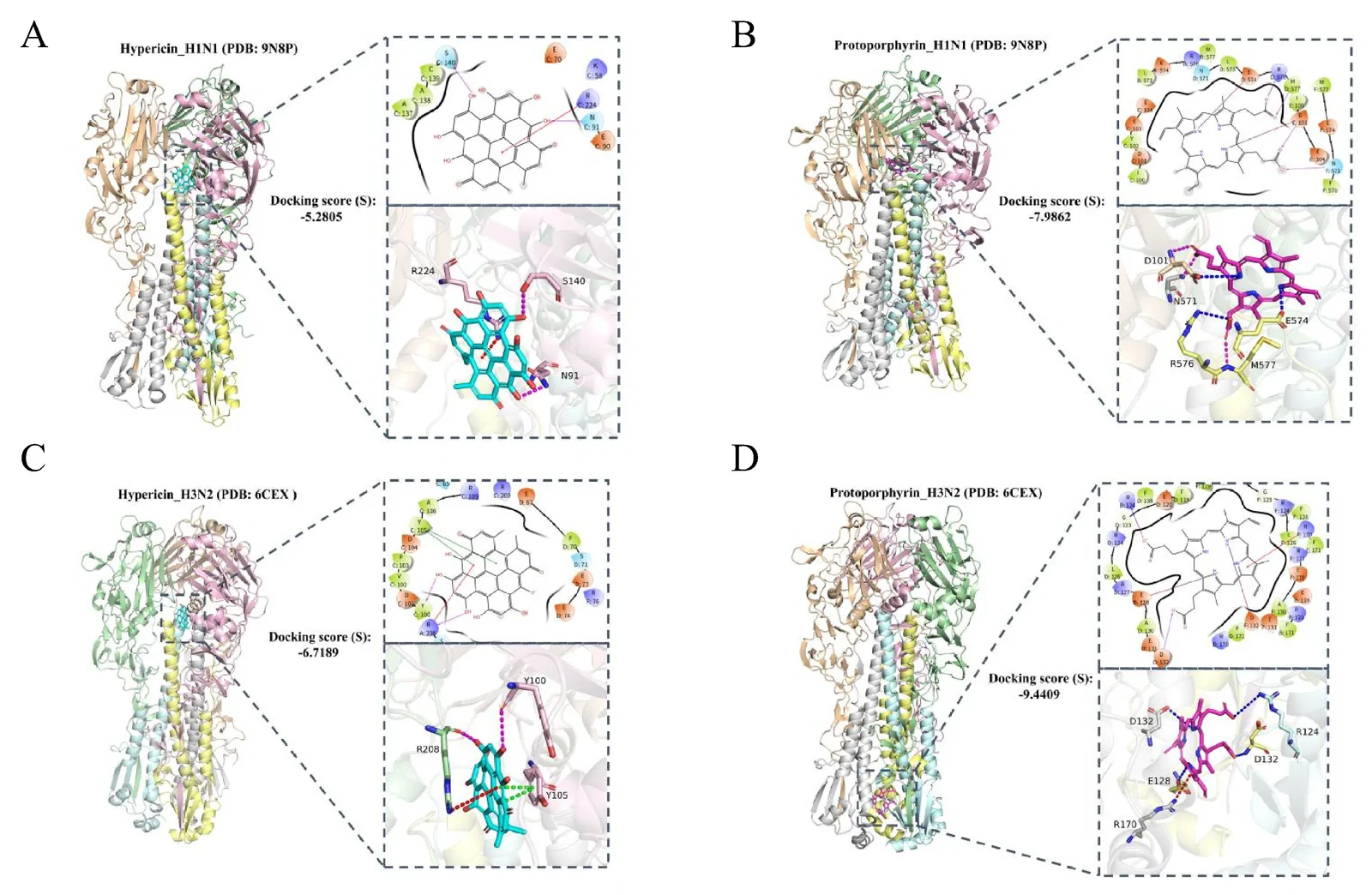
Molecular docking simulations of compounds with HA proteins. (**A**) Hypericin docked with H1N1 HA, forming hydrogen bonds with N91 and S140, and a π–ion interaction with R224. (**B**) PPIX docked with H1N1 HA, forming hydrogen bonds with M577, D101, and N571, alongside salt bridges with E574, R576, and D101. (**C**) Hypericin docked with H3N2 HA, forming hydrogen bonds with R208 and Y100, a π–π interaction with Y105, and a π–ion interaction with R208. (**D**) PPIX docked with H3N2 HA, forming salt bridges with R124, D132, and E128, and a π–π interaction with R170.

Molecular docking studies showed that Hypericin and PPIX bind to the binding sites of both H1N1 and H3N2 HA proteins, forming key hydrogen bonds, salt bridges, and hydrophobic interactions with specific residues (Figure 9). For H1N1, Hypericin forms hydrogen bonds with N91 and S140, and a π–ion interaction with R224 (Figure 9A). PPIX forms hydrogen bonds with M577, D101, and N571, alongside salt bridges with E574, R576, and D101 (Figure 9B). For H3N2, Hypericin forms hydrogen bonds with R208 and Y100, a π–π interaction with Y105, and a π–ion interaction with R208 (Figure 9C). PPIX forms salt bridges with R124, D132, and E128, and a π–π interaction with R170 (Figure 9D). The interaction energy between the protein and the compound is evaluated using the S score, where a lower value indicates stronger binding affinity. Specifically, the S scores for the docked complexes are as follows: H1N1 + Hypericin (−5.2805), H1N1 + PPIX (−7.9862), H3N2 + Hypericin (−6.7189), and H3N2 + PPIX (−9.4409). The consistently lower S scores of PPIX indicate its stronger affinity towards both HA proteins. These structural interactions provide a structural basis for their broad-spectrum activity.

### 2.10. CPE Inhibition Assay

**Figure 10:**
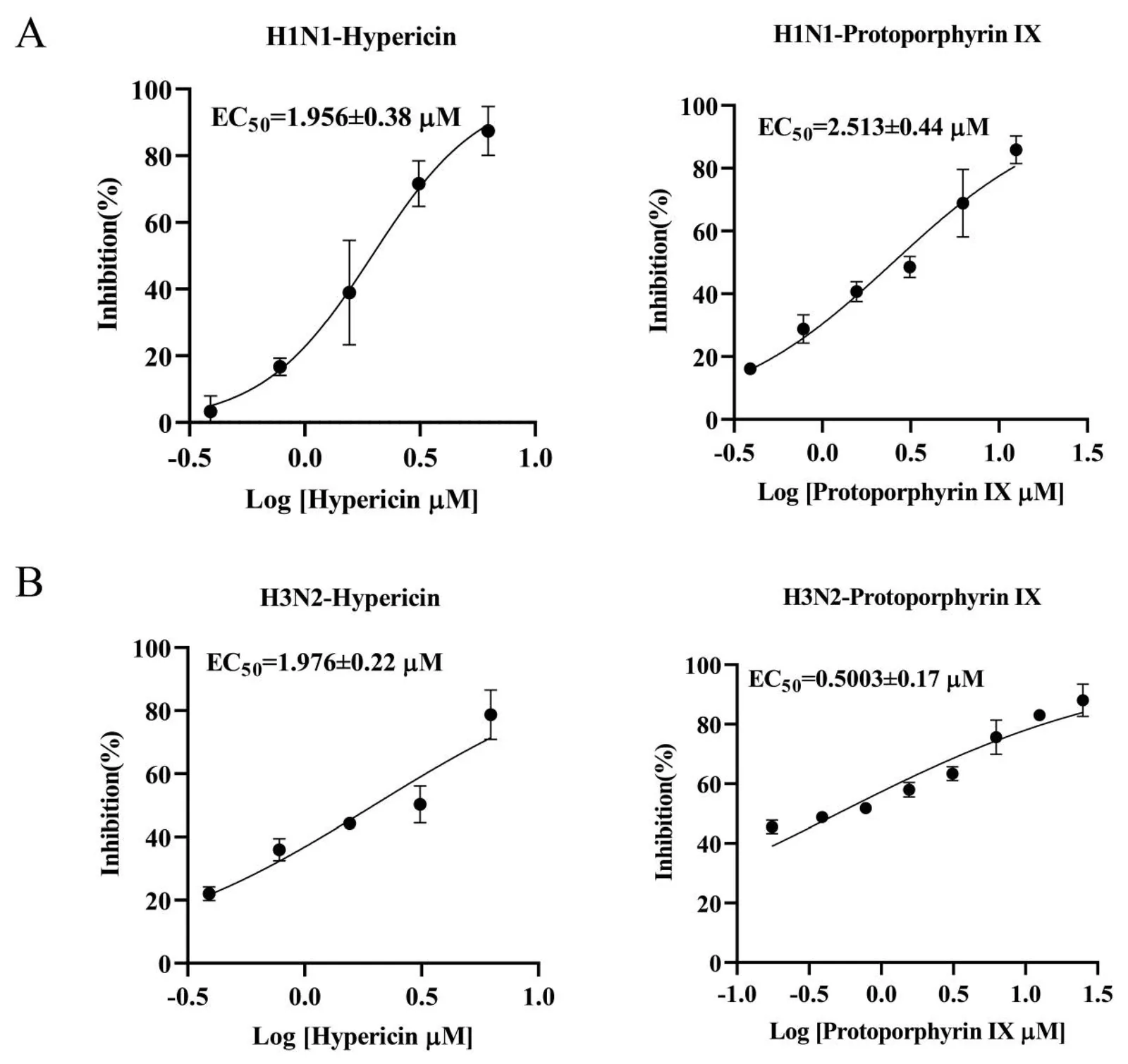
Antiviral efficacy of Hypericin and PPIX against influenza A viruses in MDCK cells. (**A**) Dose-dependent protection against virus-induced CPE for the H1N1 (A/Puerto Rico/8/1934) subtype. The EC_50_ values were determined to be 1.96 ± 0.38 μM for Hypericin and 2.513 ± 0.44 μM for PPIX. (**B**) Dose-dependent protection against the H3N2 (A/Aichi/2/1968) subtype. The EC_50_ values were 1.98 ± 0.22 μM for Hypericin and 0.50 ± 0.17 μM for PPIX. Data are presented as mean ± SD of *n* = 3 independent biological replicates.

The antiviral efficacy of the two hit compounds was evaluated using a CPE inhibition assay in MDCK cells infected with Influenza A H1N1 (A/Puerto Rico/8/1934) and Influenza A H3N2 (A/Aichi/2/1968). Both Hypericin and PPIX exhibited robust, dose-dependent protection of MDCK cells from virus-induced cytopathic effects. For the H1N1 subtype, the EC_50_ values were determined to be 1.96 ± 0.38 μM for Hypericin and 2.51 ± 0.44 μM for PPIX (Figure 10A). For the H3N2 subtype, Hypericin displayed an EC_50_ of 1.98 ± 0.22 μM, while PPIX showed even more potent activity, with an EC_50_ of 0.50 ± 0.17 μM (Figure 10B). Notably, PPIX was approximately four-fold more potent against H3N2 than against H1N1, indicating a subtype-dependent efficacy profile. The inhibitory concentrations obtained here are consistent with previously reported values [20,21,22]. Collectively, these results demonstrate that both compounds effectively suppress influenza virus-induced CPE at low micromolar concentrations, with PPIX showing superior activity against the H3N2 subtype, consistent with their designation as viral-side attachment inhibitors.

### 2.11. In Silico ADMET Prediction and Structural Optimization Strategies

To evaluate the drug-like properties and potential liabilities of the identified hits, we predicted the ADMET profiles of Hypericin and PPIX using ADMETlab 2.0 and SwissADME (Table 1). Hypericin exhibits severe physicochemical limitations. It possesses a high molecular weight (504.08 Da), extreme lipophilicity (LogP = 7.13), and exceptionally poor aqueous solubility (LogS = −10.19). Consequently, it violates Lipinski’s Rule of Five and demonstrates low Caco-2 and MDCK permeability, predicting compromised oral absorption. Pharmacokinetically, Hypericin is predicted to inhibit CYP1A2 and CYP2C9, raising the risk of drug–drug interactions. Notably, it shows a high probability of drug-induced liver injury (DILI, 0.984), skin sensitization, and eye irritation. PPIX also presents significant developmental challenges. With a molecular weight of 562.26 Da and a LogP of 6.79, it fails the Lipinski and GSK rules. While its solubility (LogS = −2.36) is superior to Hypericin, PPIX suffers from exceedingly low intestinal absorption (HIA probability = 0.012) and poor bioavailability (F < 30% = 0.003). Its distribution profile is further limited by exceptionally high plasma protein binding (PPB = 97.62%) and a low unbound fraction (Fu = 1.55%). Toxicity predictions indicate that PPIX carries a high risk of DILI (0.969) and respiratory toxicity (0.974), and triggers multiple genotoxic carcinogenicity alerts (3 alerts). Additionally, it is a potent inhibitor of CYP1A2 (0.94).

## 3. Discussion

In this study, we established a cell-based ELISA HTS platform to discover broad-spectrum influenza entry inhibitors. Its core innovation is the strategic use of HRP-conjugated SNA as a functional surrogate for diverse HA subtypes. Although SNA (a type-2 ribosome-inactivating protein with lectin activity [23]) and HA (a trimeric class I viral fusion protein [24]) belong to distinct protein families, both specifically recognize α2,6-linked sialic acids [25,26], the primary receptor for human-adapted influenza viruses [14,27]. Both recognize sialic acid through a shared biophysical logic, in which aromatic residues (Tyr/Trp) anchor its nonpolar face via CH–π interactions while polar or charged residues (His, Asp, Asn, Arg) lock its carboxylate, glycerol hydroxyl and acetamido group through hydrogen bonds and salt bridges, a strategy conserved even in the structurally unrelated siglecs, where a single arginine is essential for sialic acid binding [28]. The α2,6-sialic acid specificity of the SNA-I B-chain binding pocket is well documented [12,13]. The SNA-II crystal structure (PDB: 3CA5)**,** which shares the ricin-B-type β-trefoil fold of SNA-I, further reveals that a conserved aspartate coordinates the O3/O4 atoms of the galactose moiety of the LacNAc core [29]. On the HA side, the RBS adheres to the same logic. Its conserved floor (Tyr98, Trp153, His183, Tyr195; H3 numbering, corresponding to Tyr95 in H1 numbering) and the surrounding 130-loop, 150-loop, 190-helix, and 220-loop engage sialic acid in the same manner, with Tyr98 hydrogen-bonding the glycerol 8-OH, Trp153 forming a CH–π interaction with the N-acetyl methyl, and the 130-loop anchoring the carboxylate [30,31]. Crucially, this receptor-binding framework and its core residues are conserved across influenza A subtypes and influenza B lineages, even as the surrounding antigenic surface of the globular head undergoes continuous drift, which is precisely what makes the receptor-recognition step a subtype-independent vulnerability that can be targeted by a single surrogate probe [31]. To confirm that this shared recognition logic translates into a functional readout, we verified that the two probes engage the same receptor substrate. A single probe can therefore report the conserved receptor-recognition step across subtypes, rendering this BSL-1 platform highly scalable and quantitative while effectively bypassing antigenic drift. A further caveat is inherent to the surrogate itself. SNA is a lectin rather than HA, so the assay alone cannot tell whether a compound blocks receptor engagement or instead interferes with the SNA probe. That possibility is excluded by the SNA-free HAI and SPR assays performed here for every reported hit, neither of which relies on the lectin probe at all. The non-enzymatic, cell-level flow cytometry readout of the same receptor engagement corroborates this conclusion, and because none of these readouts depends on the HRP/TMB chemistry, their concordance indicates that both compounds interact with the virion rather than with the SNA probe. Two limitations must nevertheless be stated explicitly. First, no virucidal assay or fusion-blocking assay was performed, so the readouts used here cannot exclude either contribution. HAI reports only virion bridging to erythrocytes while SPR reports only binding to the immobilized HA ectodomain, and neither can separate blockade of the HA receptor-binding site from virion damage or from inhibition of a post-attachment step such as fusion. This absence is a limitation of the present study. The mechanism is therefore reported as viral-side attachment inhibition rather than as a defined blockade of the HA receptor-binding site. Second, no broadly accepted, validated HA-RBS reference standard is available, and the sensitivity of the platform to bona fide HA-specific blockers has therefore not been definitively calibrated. The platform should accordingly be regarded as a presumptive screen whose hits require orthogonal confirmation, which was applied here to every reported hit.

With the surrogate validated at the receptor level and the interpretation of a positive signal defined, we applied the platform to a collection of 2600 compounds and identified Hypericin and PPIX as influenza virus entry inhibitors. The readouts proved mutually consistent, but because the SPR affinities are low (KD in the 10^−4^–10^−3^ M range), this weak direct interaction alone cannot account for the potent antiviral efficacy observed in the CPE assays. Docking indicates that both compounds target the canonical HA RBS framework in distinct modes, with Hypericin mapping to the RBS boundaries as a competitive blocker, whereas PPIX blocks the RBS periphery in H3N2 and additionally engages the HA1/HA2 interface in H1N1. These findings suggest a unified, concentration-dependent dual-pronged mechanism, in which the compounds bind HA directly and block receptor recognition at lower concentrations (potentially locking the prefusion conformation in the case of PPIX), whereas at higher concentrations their large, planar, conjugated scaffolds may integrate into the viral envelope and exert the membrane-targeting virucidal activity previously described for this compound class [21]. HA is, however, only one interaction partner, and the full antiviral potency of both compounds is multimodal. It should be emphasized that this study is framed as the development and validation of a screening platform rather than as a comprehensive mechanistic elucidation of the two hits, and the dual-pronged model outlined above is a working hypothesis. Hypericin and PPIX are accordingly designated viral-side attachment inhibitors with weak HA binding and multimodal antiviral activity. Independent of this mechanistic complexity, the developability of the two scaffolds is limited in its own right, and ADMET profiling revealed substantial druggability challenges. These liabilities position the molecules as pharmacological tools for dissecting entry mechanisms rather than as immediate clinical candidates, and rational structural optimization would be required for clinical translation.

Beyond the properties of the hits themselves, the usefulness of the platform rests on how reliably its readout reflects target engagement rather than an assay artifact, and in this format the principal route to a false-positive signal is interference with the detection chemistry rather than with the target. The specific liabilities are (i) direct inhibition of the HRP reporter; (ii) redox cycling that consumes H_2_O_2_ or reduces TMB non-enzymatically; (iii) intrinsic absorbance, autofluorescence or optical quenching in the 450 nm window, a liability that preferentially accompanies conjugated heterocyclic and porphyrin-like chromophores [32,33]; (iv) detergents and membrane-active amphiphiles that cause cell detachment and thereby lower the signal; and (v) aggregation-based inhibition [33,34]. Both hits here, Hypericin (a naphthodianthrone) and PPIX (a porphyrin), belong to class (iii), so we did not rest the conclusion on the colorimetric readout alone. Three independent lines of evidence exclude such an artifact. Neither compound inhibited HRP in the compound/probe/substrate control. Both reproduced dose-dependent inhibition in the orthogonal FITC-SNA flow cytometry readout, with IC_50_ values in close agreement with the ELISA, and SPR, a label-free readout intrinsically insensitive to compound color, confirmed direct, dose-dependent binding to H1N1 and H3N2 HA. Consistent with this, PAINS substructure filtering returned no alerts for either hit. Having considered false positives, we note that false negatives are equally relevant for users of the platform. Because the model uses ST6GAL1-overexpressing MDCK cells enriched in α2,6-linked sialic acids, compounds selective for α2,3-linked receptors are not expected to register. Because the probe reports receptor recognition specifically, HA binders acting outside the receptor-binding site, for example at allosteric or fusion-related epitopes, fall outside the readout. Finally, with a single screening concentration of 25 μM and a 50% inhibition threshold, genuine but weak binders are missed by design, as illustrated by the low-affinity positive control 6′-SL. Together with the composition of the collection, these considerations define the chemical space and practical limits of the platform and provide guidance for its use with larger, more diverse libraries.

## 4. Materials and Methods

### 4.1. Reagents

Sambucus nigra agglutinin (SNA, Cat: Q-WS1027) and 4% paraformaldehyde (PFA, Cat: BL539A) were sourced from Qiyuebio^TM^ (Xi’ an, China) and biosharp (Nanjing, China) respectively. Dimethyl sulfoxide (DMSO, Cat: ST038-500ml) was obtained from Beyotime (Shanghai, China), while 6’-sialyllactose (6’-SL, Cat: B23777) was purchased from Shanghai Yuanye Bio-Technology (Shanghai, China). Hypericin (Cat: HY-N0453) and Protoporphyrin IX (PPIX, Cat: HY-B1247) were obtained from MCE (Monmouth Junction, NJ, USA). Bioshark (Shanghai, China) provided the 96-well culture plates (Cat: BS-MP-96W-CL), and the 1% guinea pig red blood cells (GPRBC, Cat: SBJ-RBC-GP001-100ml) were acquired from Nanjing sbjbio (Nanjing, China). Recombinant HA proteins of influenza A H1N1 (A/Puerto Rico/8/1934; Cat: 11684-V08H3), H3N2 (A/Aichi/2/1968; Cat: 11707-V08H), and H3N2 (A/Brisbane/10/2007; Cat: 11056-V08H), together with HRP-conjugated monoclonal antibodies against H1N1 (A/Puerto Rico/8/1934) HA (Cat: 11684-MM03-H) and H3N2 (A/Brisbane/10/2007) HA (Cat: 11056-MM01-H), were all purchased from Sino Biological (Beijing, China). To generate the detection probe, HRP (Cat: P8020, Solarbio, Beijing, China) was conjugated to SNA using a commercial labeling kit (Cat: ab102890 1 mg, Abcam, Cambridge, UK) to produce HRP-conjugated SNA (HRP-SNA). ELISA stop solution (Cat: BL1171A) and CCK8 (Cat: K1018-5ml) were sourced from biosharp (Nanjing, China) and APExBio (Houston, TX, USA), respectively. The Madin–Darby Canine Kidney (MDCK) cell line and MDCK cells with high expression of α-2,6-linked sialic acid receptors deposited at the China General Microbiological Culture Collection Center (CGMCC) under the accession number CGMCC No. 46791 are all maintained within our laboratory. Additionally, a microbial natural product and clinical-stage anti-infective compound library (stored as 10 mg/mL stock solutions in DMSO; sourced from TargetMol (Boston, MA, USA) and Selleck (Houston, TX, USA)) was provided by the Institute of Medicinal Biotechnology, Chinese Academy of Medical Sciences (CAMS) and Peking Union Medical College (PUMC). FITC-conjugated SNA (Cat: Q-0002983, Xi’an Qiyue, Xi’ an, China) was used for the flow cytometric analysis.

### 4.2. Binding Assay of Influenza HA and HRP-SNA on MDCK-ST6GAL1 Receptor

To directly compare the receptor recognition of influenza HA and SNA on the same cellular receptor, MDCK-ST6GAL1 cells were seeded into 96-well plates (1 × 10^5^ cells/well), cultured for 24 h and fixed with 4% PFA at 37 °C for 15 min. In a separate set of wells, either purified recombinant HA representative of the two major human influenza A subtypes, H1N1 HA (A/Puerto Rico/8/1934) or H3N2 HA (A/Brisbane/10/2007), or HRP-SNA was incubated with the fixed monolayers at 37 °C for 60 min. In a parallel competition arm, either recombinant HA or HRP-SNA was incubated with the fixed monolayers in the presence of increasing concentrations of 6′-SL (the known positive compound; 0.78–200 μM) under otherwise identical conditions, and IC_50_ values were derived from the corresponding dose–response curves. Bound H1N1 HA was detected with an HRP-conjugated anti-HA monoclonal antibody, while bound SNA was detected directly through its conjugated HRP. All subsequent steps, including five PBS washes, TMB substrate development for 15 min, addition of stop solution and OD_450_ measurement, were performed identically across all wells. All conditions were tested in triplicate.

### 4.3. Establishment and Optimization of the Cell-Based ELISA Screening Model

#### 4.3.1. Establishment of the Cell-Based ELISA Screening Model

MDCK-ST6GAL1 cells were used to establish the screening platform for influenza virus entry inhibitors. Cells were seeded into 96-well plates at a density of 1 × 10^5^ cells/well and cultured for 24 h until reaching approximately 80% confluence. The cells were then incubated with HRP-SNA to specifically label the surface receptors. Following incubation, cells were washed five times with PBS to remove unbound probes. Subsequently, 100 μL of TMB substrate was added to each well and incubated at 37 °C for 15 min in the dark. The reaction was terminated by adding 50 μL of stop solution, and the optical density at OD_450_ was measured using a microplate reader (PerkinElmer, Inc. (Revvity), Waltham, MA, USA).

#### 4.3.2. Optimization of the Cell-Based ELISA Screening Model

Critical parameters were systematically optimized to ensure optimal assay sensitivity and stability. The optimal HRP-SNA concentration was determined by incubating cells with the lectin at concentrations ranging from 0.1 to 20 μg/mL. The necessity of cell fixation was evaluated by comparing cells treated with 100 μL of 4% PFA at 37 °C for 15 min against unfixed controls. For blocking optimization, cells were treated with 50 μL of 5% BSA-PBS for 1 h or left unblocked. Incubation times (30, 45, or 60 min) and temperatures (4 °C, 25 °C, or 37 °C) were also varied to identify optimal binding conditions. Additionally, to assess compatibility with small molecule libraries, DMSO tolerance was evaluated by pre-incubating cells with DMSO concentrations ranging from 0% to 10% for 60 min. Following each specific treatment, all optimization groups underwent the standard procedure of PBS washing, TMB substrate reaction, and absorbance measurement as described in Section 4.3.1 to finalize the optimal conditions.

### 4.4. Assay Validation and Determination of Z’ Factor

To validate the applicability of the constructed screening model, 6’-SL was used to assess the reproducibility of the assay. Independent concentration series (0.78–200 μM) were each performed on a different day with independently prepared reagents, following the seeding, fixation, competition and detection procedure described in Section 2.2. DMSO-treated wells and wells without HRP-SNA served as negative and blank controls, respectively. IC_50_ values were derived from the corresponding dose–response curves, and the inter-assay coefficient of variation (CV) was calculated from the independent series. Finally, the robustness of the optimized assay was evaluated by calculating the Z’ factor using the data from the positive and negative controls.

### 4.5. Inhibitor Screening and IC_50_ Determination

For the primary screening of the compound library, the optimized cell-based ELISA model was employed at a single concentration of 25 μM. Positive control wells were established using MDCK-ST6GAL1 cells treated with 6’-SL followed by HRP-SNA. Negative control wells (representing maximal binding) consisted of cells treated with HRP-SNA in the absence of inhibitors. Positive and negative control wells were included on every screening plate, and the inhibition rate of each test well was normalised to the control wells on the same plate with the same fixed hit threshold applied to all plates. Compounds exhibiting an inhibition rate greater than 50% at 25 μM were selected as primary hits. To eliminate false positives arising from cytotoxicity, these hits were subsequently evaluated for cellular toxicity using the CCK-8 assay. Compounds demonstrating low cytotoxicity were further subjected to dose–response analysis to determine their half-maximal inhibitory concentration (IC_50_) values using serial dilutions by plotting the inhibition rate against the logarithm of the compound concentration and fitting the data using a dose–response curve. The inhibition rate was calculated using the following formula:

$$Inhibition\;Rate\;(\%)=(1-\frac{{OD}_{sample}-{OD}_{blank}}{{OD}_{negative\;control}-{OD}_{blank}})\times 100\%$$

### 4.6. Exclusion of Compound Interference with the Detection System

To ensure the reliability of the screening data, potential interference from the compounds with the detection system was evaluated. Different concentrations of the compounds were pre-incubated with HRP-SNA (1 μg/mL) at 37 °C for 30 min to assess direct inhibition of HRP activity. Following the addition of TMB substrate and a 15 min incubation, the reaction was stopped, and the absorbance at 450 nm was compared to a control group lacking compounds. Because this reaction mixture contains the compounds together with the HRP-SNA probe and the TMB substrate but no cells, the comparison captures both the compounds’ own absorbance at 450 nm (their optical background) and any effect of the compounds on the HRP/TMB chemistry.

### 4.7. Flow Cytometry Validation of Receptor–Ligand Binding

To validate the ELISA readout independently, receptor–ligand binding was analyzed by flow cytometry, an approach previously established for the quantitative assessment of influenza virus binding to cell-surface receptors [13]. FITC-conjugated SNA (1 μg/mL) was pre-incubated with compounds (Hypericin, PPIX, 2 compounds that scored negative in the primary screen, and the positive control (6′-SL)) or with vehicle (0.1% DMSO) for 1 h at 37 °C, after which MDCK-ST6GAL1 cells were added. The preference of SNA for α2,6- over α2,3-linked sialic acid is well established [12,35], and MDCK-ST6GAL1 cells were chosen specifically to enrich this determinant. Cells were grown to 90% confluence in 6-well plates, detached with PBS-EDTA to preserve surface sialic acid, and resuspended in PBS containing 1% BSA. Aliquots of 1 × 10^5^ cells were added to the pre-formed probe–compound mixtures and incubated for 1 h at 4 °C, a temperature chosen to prevent receptor internalization and lectin recycling. To control for the intrinsic fluorescence of the two compounds, compound-only samples (cells plus compound, no probe) and vehicle-only samples (cells plus vehicle, no probe) were acquired in parallel and used for background subtraction. After two washes, at least 10,000 viable single-cell events per sample were acquired on a BD FACSVerse using the FITC channel (gating: FSC/SSC singlets → viable cells) and analyzed in CytExpert 2.6. The inhibition rate was derived from the integrated mean fluorescence intensity (iMFI) as [1 − (iMFI_compound_ − iMFI_blank_)/(iMFI_FITC-SNAcontrol_ − iMFI_blank_)] × 100%, where iMFI = % Parent × MFI. Dose–response curves for the two hits (3.12–100 μM) were fitted to derive IC_50_ values. All samples were analyzed in triplicate in three independent experiments and data are presented as mean ± SD.

### 4.8. Hemagglutination Inhibition (HAI) Assay

To distinguish whether the inhibitors target the viral HA or the cellular sialic acid receptors, HAI assays were performed using 1% GPRBCs with modified incubation sequences [36]. Viral HA titers were first standardized to ensure optimal assay sensitivity. To evaluate direct binding to viral particles, the test compounds were pre-incubated with the influenza virus (HA) prior to the addition of GPRBCs. Conversely, to assess potential interference with the cell surface receptors, the compounds were pre-incubated with GPRBCs before the introduction of the influenza virus (HA). In both setups, the sedimentation of erythrocytes into a compact pellet, as opposed to a diffuse agglutination pattern, indicated the inhibition of hemagglutination. Because intact, replication-competent virions are used, hemagglutination in this assay reflects virion–erythrocyte bridging by surface HA rather than binding of the isolated protein.

### 4.9. Surface Plasmon Resonance (SPR) Analysis

The binding kinetics between the candidate compounds and purified influenza HA proteins (H1N1 and H3N2 subtypes) were characterized using SPR spectroscopy (Reichert 2SPR, Buffalo, NY, USA). The running buffer consisted of 1 × PBS containing 0.05% Tween™ 20 and 5% DMSO. Recombinant HA proteins were immobilized onto a CM5 gold biosensor chip following activation with ethanolamine buffer (1 mol/L, pH 8.0). Serial dilutions of the compounds were injected over the protein-coated sensor surface at a flow rate ensuring a contact time of 90 s, followed by a dissociation phase of 330 s. Real-time refractive index units (RIUs) were monitored, and the data were converted to resonance units (RUs) for analysis. The association rate constant (K*on*) and dissociation rate constant (K*off*) were calculated using TraceDrawer software (version 1.7.1, Ridgeview Instruments, Uppsala, Sweden). The equilibrium dissociation constant (K*_D_*) was subsequently derived from the ratio of K*off* to K*on*. A parallel reference channel was derivatized with an equal amount of BSA, and the identical concentration series of each compound was injected over both the HA and the BSA surfaces to assess nonspecific surface binding.

### 4.10. Cytopathic Effect (CPE) Inhibition Assay

The antiviral efficacy of the compounds was evaluated based on the inhibition of virus-induced CPE in MDCK cells [37]. Cells were pre-treated with serial dilutions of the compounds for 1 h prior to infection with influenza virus at a multiplicity of infection (MOI) of 0.1. Following a 48 h incubation period, cell viability was quantified using the CCK-8 assay. The 50% effective concentration (EC_50_) was calculated from dose–response curves, and the selectivity index (SI) was determined as the ratio of the 50% cytotoxic concentration (CC_50_) to the EC_50_.

### 4.11. Molecular Docking Simulation

Computational docking studies were performed to investigate the potential binding modes of the compounds. Crystal structures of H1N1 HA (PDB: 9N8P) [38] and H3N2 HA (PDB: 6CEX) [39] were prepared using the QuickPrep module in MOE software 2024 (2024.0601) under the AMBER10: EHT force field. The preparation process included protonation, hydrogen addition, energy minimization to eliminate steric clashes, and side-chain optimization. Ligand structures of Hypericin and Protoporphyrin IX were processed using the LigPrep module in Schrödinger software 2023-1, which included hydrogen addition, protonation, tautomer generation, and energy minimization for conformational optimization. As no specific binding sites were predefined, the entire protein surface was designated as the docking region. Docking simulations were executed using the MOE Dock module, employing the Triangle Matcher algorithm for pose generation and the London dG scoring function to evaluate the binding affinity of each pose. The top 300 docking poses with optimal conformations were retained, with a maximum of 50 conformations outputted for subsequent analysis.

### 4.12. Absorption, Distribution, Metabolism, Excretion, and Toxicity (ADMET) Prediction

The ADMET profiles of the active compounds were predicted using the online platform ADMETlab 3.0. Structural inputs in SMILES format were generated using ChemDraw Ultra 14.0.

### 4.13. Statistical Analysis

Quantitative data are expressed as the mean ± standard deviation (SD) from at least three independent experiments. Statistical differences were analyzed by one-way analysis of variance (ANOVA) followed by Tukey’s post hoc test using GraphPad Prism 9.0 software. Significance levels were defined as * *p* < 0.05, ** *p* < 0.01, *** *p* < 0.001, and **** *p* < 0.0001. All experiments were performed with at least three independent biological replicates, each comprising technical triplicate wells.

## 5. Conclusions

This work delivers a practical screen for the subtype-independent receptor-recognition step, removing a long-standing obstacle to the discovery of broad-spectrum entry inhibitors. SNA serves as a functional surrogate for HA rather than a structural one. This functional equivalence is sufficient for the assay to detect compounds that interfere with the conserved binding event. The readout, however, follows receptor recognition rather than HA engagement itself, so site-specific blockade, membrane-mediated damage and virucidal action remain indistinguishable here. Independent confirmation is therefore integral to the workflow rather than optional, as implemented here by flow cytometry, HAI, SPR and CPE assays. On this evidence, Hypericin and PPIX are best regarded as viral-side attachment inhibitors with weak HA binding and multimodal antiviral activity.

## Figures and Tables

**Table 1:** In silico ADMET prediction of Hypericin and PPIX.

Category	Property	Hypericin	PPIX	Comment/Optimal Range
**Physicochemical**	MW (g/mol)	504.08	562.26	Optimal: 100–600
	LogP	7.132	6.789	Optimal: 0–3
	LogS	−10.19	−2.359	Optimal: −4 to 0.5
	TPSA (Å^2^)	155.52	131.96	Optimal: 0–140
	H-bond Acceptors	8	8	Optimal: 0–12
	H-bond Donors	6	4	Optimal: 0–7
	Rotatable Bonds	0	8	Optimal: 0–11
**Absorption**	Caco-2 Permeability (log cm/s)	−5.387	−5.33	Optimal: >−5.15
	MDCK Permeability (cm/s)	4 × 10^−6^	1.1 × 10^−5^	Low: <2 × 10^−6^; Med: 2–20 × 10^−6^
	HIA (Probability)	0.995 (HIA+)	0.012 (HIA−)	Category 1: HIA+ (>30% absorbed)
	F30% (Probability)	1.0 (F30%+)	0.003 (F30%−)	Category 1: Bioavailability < 30%
**Distribution**	PPB (%)	79.54	97.62	Optimal: <90%
	BBB Penetration (Probability)	0.001 (BBB−)	0.001 (BBB−)	Category 1: BBB+
	VD (L/kg)	0.429	0.316	Optimal: 0.04–20
	Fu (%)	21.74	1.553	High: >20%; Low: <5%
**Metabolism**	CYP1A2 Inhibitor (Prob.)	0.768	0.94	Category 1: Inhibitor
	CYP2C9 Inhibitor (Prob.)	0.643	0.454	Category 1: Inhibitor
	CYP2D6 Inhibitor (Prob.)	0.001	0.339	Category 1: Inhibitor
	CYP3A4 Inhibitor (Prob.)	0.006	0.1	Category 1: Inhibitor
**Excretion**	Clearance (mL/min/kg)	0.561	0.849	Low: <5; Med: 5–15; High: >15
	T1/2 (Probability)	0.264	0.448	Category 1: Long half-life (>3 h)
**Toxicity**	DILI (Probability)	0.984	0.969	Category 1: High risk of DILI
	AMES Toxicity (Probability)	0.099	0.049	Category 1: Ames positive
	hERG Blocker (Probability)	0.001	0.035	Category 1: Active
	Skin Sensitization (Prob.)	0.951	0.057	Category 1: Sensitizer
	Eye Irritation (Probability)	0.959	0.007	Category 1: Irritant
	Respiratory Toxicity (Prob.)	0.05	0.974	Category 1: Toxicant
	Carcinogenicity (Probability)	0.052	0.476	Category 1: Carcinogen
**Drug-likeness**	Lipinski Rule	Rejected	Rejected	MW ≤ 500; LogP ≤ 5
	QED	0.123	0.225	Attractive: >0.67
	PAINS Alerts	0	0	0 alerts is preferred

## Data Availability

The original contributions presented in this study are included in the article. Further inquiries can be directed to the corresponding authors.
